# Advances and insights in the diagnosis of viral infections

**DOI:** 10.1186/s12951-021-01081-2

**Published:** 2021-10-30

**Authors:** Julija Dronina, Urte Samukaite-Bubniene, Arunas Ramanavicius

**Affiliations:** 1 Laboratory of Nanotechnology, Department of Functional Materials and Electronics, Center for Physical Sciences and Technology, Sauletekio av. 3, Vilnius, Lithuania; 2 Department of Physical Chemistry, Faculty of Chemistry and Geoscience, Vilnius University, Naugarduko str. 24, 03225 Vilnius, Lithuania

**Keywords:** COVID-19, SARS-CoV-2 virus detection, Antibody-antigen complex, Immunosensors, Polymerase chain reaction (PCR), CRISPR-Cas for DNA-Sensors, Biosensors, Surface plasmon resonance (SPR), Photoluminescence

## Abstract

Viral infections are the most common among diseases that globally require around 60 percent of medical care. However, in the heat of the pandemic, there was a lack of medical equipment and inpatient facilities to provide all patients with viral infections. The detection of viral infections is possible in three general ways such as (i) direct virus detection, which is performed immediately 1–3 days after the infection, (ii) determination of antibodies against some virus proteins mainly observed during/after virus incubation period, (iii) detection of virus-induced disease when specific tissue changes in the organism. This review surveys some global pandemics from 1889 to 2020, virus types, which induced these pandemics, and symptoms of some viral diseases. Non-analytical methods such as radiology and microscopy also are overviewed. This review overlooks molecular analysis methods such as nucleic acid amplification, antibody-antigen complex determination, CRISPR-Cas system-based viral genome determination methods. Methods widely used in the certificated diagnostic laboratory for SARS-CoV-2, Influenza A, B, C, HIV, and other viruses during a viral pandemic are outlined. A comprehensive overview of molecular analytical methods has shown that the assay's sensitivity, accuracy, and suitability for virus detection depends on the choice of the number of regions in the viral open reading frame (ORF) genome sequence and the validity of the selected analytical method.

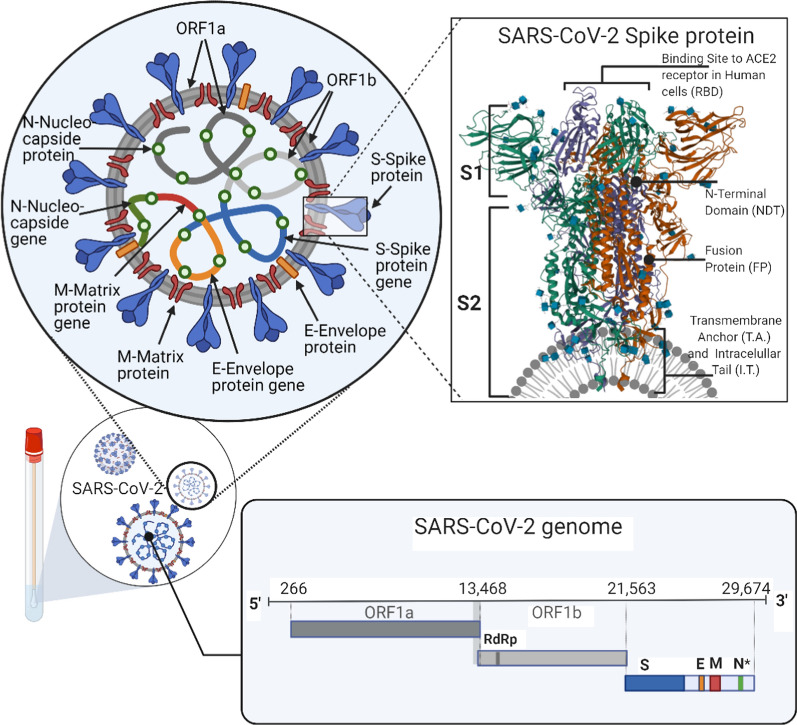

## Main

**Fig. 1:**
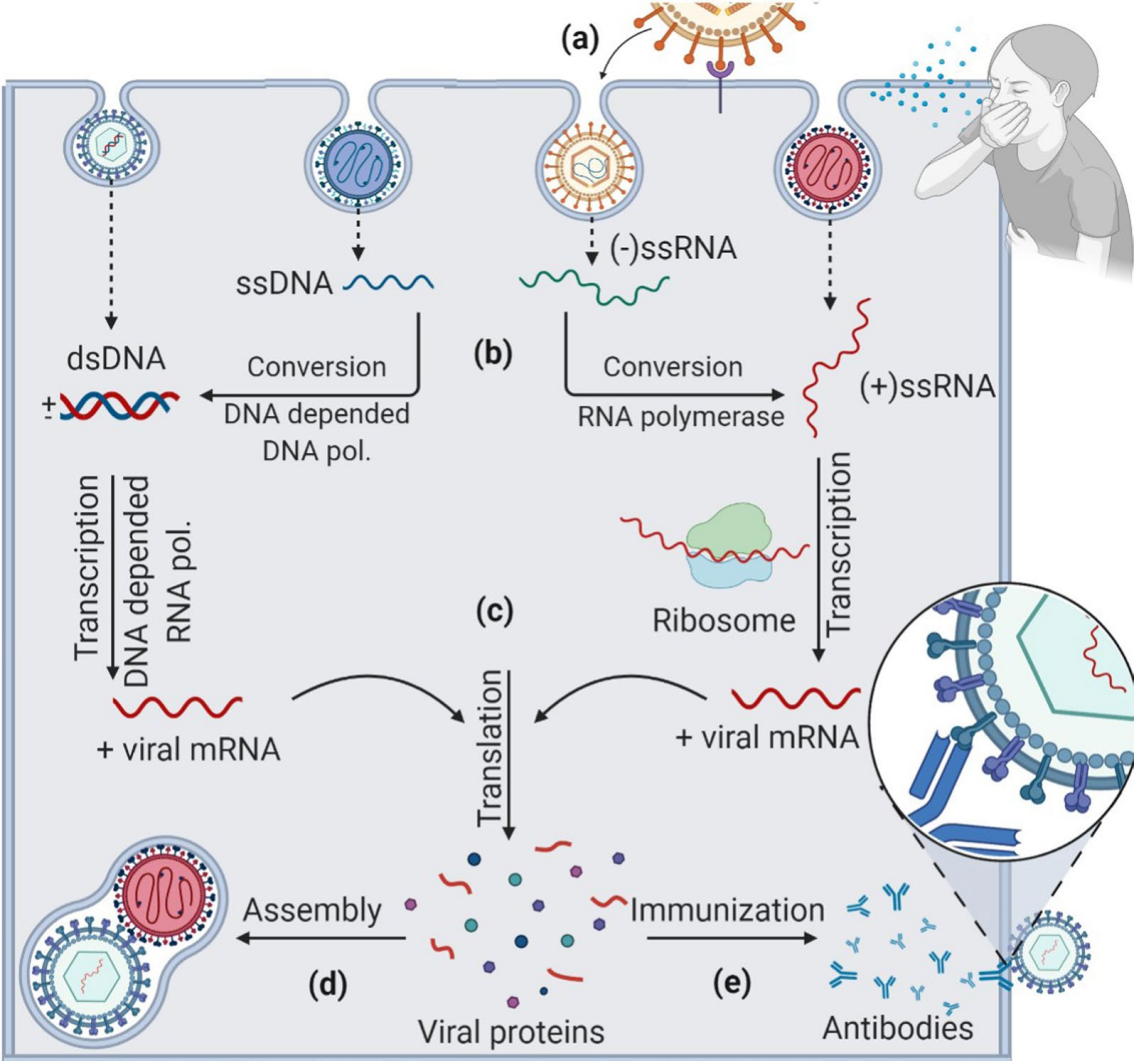
The course of viral infection in the host cell. Viral infection in the human body begins when viral hemagglutinin protein (HA) (**a**) binds to a glycolipid receptor on the cell surface. It promotes the fusion of viral cell membranes with the host cell. Once a virus enters viral genetic material (**b**) into the host cell, its replicates, and mRNA (**c**) is synthesized and converted to viral proteins. RNA viruses (like flu, SARS-CoV-2) can use their RNA to directly create countless new viruses in the host cell. DNA viruses are always making RNA copies, but rarely reverse process is occuring. Except for some retroviruses (HIV/AIDS), they copy their RNA into DNA. mRNA takes over the cell’s protein-making machinery to rapidly build a new amount of viruses. Subsequently, the synthesized viral genetic material and proteins are assembled (**d**) to form virions that help bud with neuraminidase (NA) and separate from the host cell. At the same time, the immunization takes place in the cell (**e**), the host cell begins to produce antibodies against the virus

Viral diseases are infections caused by different types of viruses. Viruses are structures of various microscopic sizes (from 20 to 900 nm) and morphological forms, composed of genetic material, which can be positive or negative sense, single (ss) or double-stranded (ds) deoxyribonucleic acid (DNA) or ribonucleic acid (RNA), surrounded by a coating based on proteins, glycoproteins, or lipids [1]. Viruses themselves do not produce energy, do not increase and have a straightforward structure. Therefore, they can only grow in other living cells (host cells), suitable for hosting a particular virus type. Once a virus enters a cell (Fig. 1), it releases and integrates its genetic material within the host cell's genome and takes over these cell's functions, which are required for the proliferation of the virus. Besides, some infected cells are proliferating themselves and, at the same time, are multiplying the genome of the virus. When the host organism's immune system detects a virus, it starts to react in a particular way. One of such ways in mammalian organisms is producing specific antibodies that help neutralize the virus and the cells that are infected by the virus. Still, the host's organism is not always able to defend itself. However, it is essential to consider that all viruses develop very rapidly and are spread by the worldwide migration of living organisms.

Viruses constantly attack humankind. Over the last two centuries, a viral pandemic has posed an increasing threat to public health worldwide. From 1889 to the present, there have been several viral pandemics throughout human history. 1889–1890 the Asiatic flu pandemic killed about 1 million people worldwide [2, 3]. 1918–1920 the Spanish flu pandemic infected 20 to more than 50 million people [4]. Asian Flu in 1957–1968 has claimed from 500,000 to 2,000,000 human lives [5]. Since 1981 more than 85 mln Humans have been infected by Human Immunodeficiency Virus (HIV), more than 33 mln have died, and 7.1 mln People at the end of 2019 are still living with acquired immunodeficiency syndrome (AIDS) but did not know that they have HIV infection (HIV, the virus that causes AIDS) [6]. World Health Organization (WHO) on 2020 March announced the outbreak of a Coronavirus disease 2019 (COVID-19) as a global pandemic causing Severe Acute Respiratory Syndrome Coronavirus-2 (SARS-CoV-2) [7, 8]. From 2020 until 2021, there are more than 86,000,000 confirmed cases of COVID-19, and more than 1.8 million human deaths have already been identified, and unfortunately, the number of cases is still rising [9, 10].

### Pandemics evolution history and studies

The Asiatic flu pandemic of 1889–1890 was quickly and in detail defined in the media of all the affected countries (France, Italy, Spain, Germany, Great Britain, Poland, Austria, Russia), and publications were mentioning not only statistics but also the symptoms of the disease and the strategy used to manage the pandemic. The origin of the Asiatic flu pandemic virus is still unknown, but it is hypothesized that the pandemic could be caused by the H3N8 virus [11, 12] or H2N2 virus [13, 14]. The end of the one-year pandemic shows that isolating the population and restricting travel is a plausible way to fight the pandemic [15, 16] but shrinks the economies and cost people lives. The fight against Spanish flu in 1918–1920 was similar to the Asiatic flu epidemic: with no information on the virus's origin, no vaccine to protect against influenza infection, and no antibiotics to treat influenza infections [17]. Only control efforts worldwide have been limited to non-pharmaceutical interventions such as isolation, quarantine, good personal hygiene, use of disinfectants, and various social restrictions. Since 2004, tissue studies of the remains of people infected by 'Spanish flu' have been started. In the remains detected the origin of the H1N1 virus [18–21].

In the face of a pandemic, various technologies and strategies are being developed, focusing on an accurate diagnosis of a particular disease, epidemiological control security, and effective prevention of virus spread. Scientists, diagnostic, and pharmaceutical companies are working hard and collaborating to detect infections faster, more accurate, and cheaper. Therefore, it is essential to know virus sequences and molecular data to apply optimal virus detection methods. Virus detection methods must be of high quality, sensitivity, specific, and relatively simple in function. Analytical virus detection methods, general-purpose reagents, or equipment must be manufactured and validated following the regulatory compliance and quality assurance (QA) rules of the International Organization for Standardization (ISO) [22, 23]. The most critical tasks and research directions during the development of bioanalytical systems for reliable determination of viral infections are:

- the release of virus or it’s genetic information from a particular matrix type,
- the development of molecular and non-molecular virus detection methods,
- the improvement of bioanalytical methods for the determination of specific antibodies against viral proteins and other virus-related structures.

The work aims to describe the validated methods used in the world market to detect SARS-CoV-2, Influenza A, B and C, HIV, and other viruses, to determine the dependencies of the methods and results, to provide insights.

### Challenges and opportunities

With experience from previous pandemics and other infections, it is possible to respond correctly to the spread of some viruses. The global COVID-19 [24] pandemic has revealed a lack of tools and skills in treatment facilities to detect infectious diseases leading to delays in diagnosis and/or arrangement of a particular treatment for large quantities of patients. For COVID-19 detection, selected rapid commercial tests have been approved by the Emergency Use Authorization (EUA), the U.S. Food and Drug Administration (FDA) and are CE marked (CE – administrative marking that indicates conformity with health, safety, and environmental protection standards for products sold within the European Economic Area (EEA) and outside of the EEA [25]. The list of rapid tests includes antibody-antigen complex formation based detection IgG/IgM [26], multiplex polymerase chain reaction (automated polymerase chain reaction (PCR) systems that can run over 100 tests per day), isothermal polymerase chain reaction, DNA Endonuclease-Targeted CRISPR Trans Reporter technique based on CRISPR–Cas12 (DETECTR) [27], Specific High Sensitivity Enzymatic Reporter unlocking platform with CRISPR Cas12 and Cas13 enzymes (SHERLOCK) methods [28]. However, the most sensitive, widely used, but long-lasting and additional equipment-based real-time or quantitative polymerase chain reaction (real-time PCR) methods are the most commonly used to diagnose viral and other pathogen infections [29]. The determination of the SARS-Cov-2 virus by polymerase chain reaction is based on several steps:

- Viral RNA is purified from tissue or blood samples. Reverse transcription reaction is performed to obtain cDNA [30] from viral RNA.
- Genetic material is amplified by polymerase chain reaction (PCR). The data analysis is performed. The entire process takes more than 2 h [31].

Some alternative innovative technological solutions are being sought to maintain the sensitivity of real-time PCR but to shorten the time from sampling to detection. However, to eliminate the gel electrophoresis step in analytical methods, DNA fragments can be identified by DNA sensors [32–34]. Even more advanced PCR-method is based on the real-time reverses-transcriptase polymerase chain reaction (real-time RT-PCR) method is more widely used today to detect COVID-19. The technique uses complex mixtures of enzymes (DNA polymerases, reverse transcriptases, nucleotides, etc.), shortening the processing time. Virus sequence detection by real-time RT-PCR is further developed to speed up virus detection. New technological achievements enable the facilitation of molecular tests or fully automated systems to simplify testing and allow a large number of samples to be tested simultaneously. New RT-PCR–based methods are also being developed to detect multiple pathogens at the same time.

**Fig. 2:**
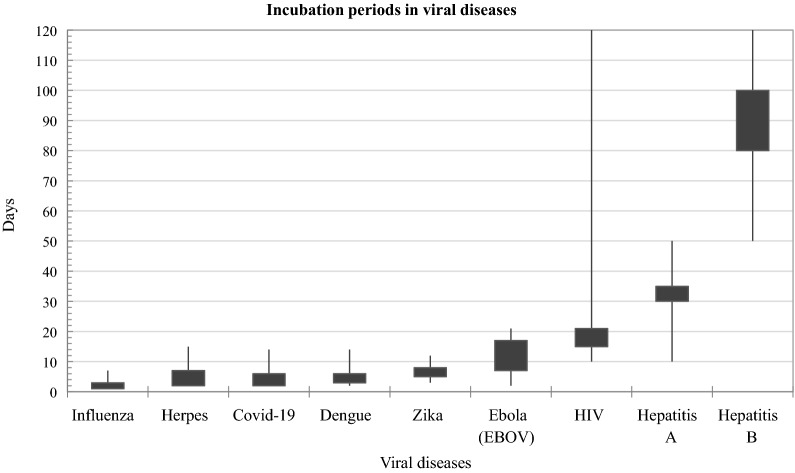
The range and the most common incubation periods in viral diseases. For influenza the most common virus incubation period in the host is 1–3 days; Herpesvirus 2–7 days; Covid-19 2–5 days [47–51]; Dengue 3–6 days [52–55]; Zika 5–8 days [56–59]; Ebola virus 7–17 days [60–63]; HIV 5–21 days; Hepatitis A 30–35 days [64, 65]; Hepatitis B 80–100 days [66–70]. In the case of COVID-19, the likelihood of detecting the virus from the respiratory tracts is likely to be 2–5 days after possible infection. Seven days after the onset of symptoms, the chance of detecting the virus directly from the respiratory tract decreases

Unfortunately, the choice of virus or disease detection method depends on many factors such as the nature of the virus, the affected area, the activity and viability time on different surfaces, or the virus incubation period (Fig. 2) [35]. As mentioned in many sources, viral diseases that directly affect the organs, such as COVID-19 viral disease does [36–38], or an infectious disease caused by the hepatitis C virus (HCV) (Hepatitis C) [39] are possibly better diagnosed using radiological images. However, the primary intention is to detect the virus in the organism as soon as possible and apply effective treatment. For example, direct amount of SARS-CoV-2 virus from the respiratory tract is exponentially dependent on the incubation period (the period from infection to onset of symptoms), and viability differs from the nature of surfaces (about 72 h on plastic, 4–8 h on copper, 8–24 h on cardboard, and 3 h on aerosol particles) as well [40, 41]. The knowledge about various virus incubation periods helps select appropriate methods for virus detection. Information on the incubation period of the virus in the host cell can help identify risk periods and local transmission and spread of the virus. For most acute infections, the virus is extracted at peak titers in the late stages of the incubation period before the host immune response has been established [42]. In COVID-19, due to the short incubation period of the virus, patients should be sampled from the upper respiratory tract (URT) and lower respiratory tract (LRT) on days 2 and 3 of symptoms, respectively, to directly detect the SARS-CoV-2 virus in the body. Extensive research has shown [43–45]. From day 3 to day 5, the amount of virus in the LRT sample increased. Here important to note that, in the URT and LRT samples, the viral load decreased from about day 7, and from day 13, the viral load was no longer detectable by RT-PCR or real-time RT-PCR. However, after 13 days, the serological antibody assay by RT-PCR or real-time RT-PCR shouldn’t be performed. Unfortunately, clinical cases of COVID-19 range are from asymptomatic to fever with mild respiratory disease, and even acute respiratory distress syndrome and death from respiratory failure or related complications [46]. When it is difficult for patients to determine the actual date of onset of infection, it would be best to follow the results of several methods.

## Molecular and non-molecular virus detection techniques

**Fig. 3:**
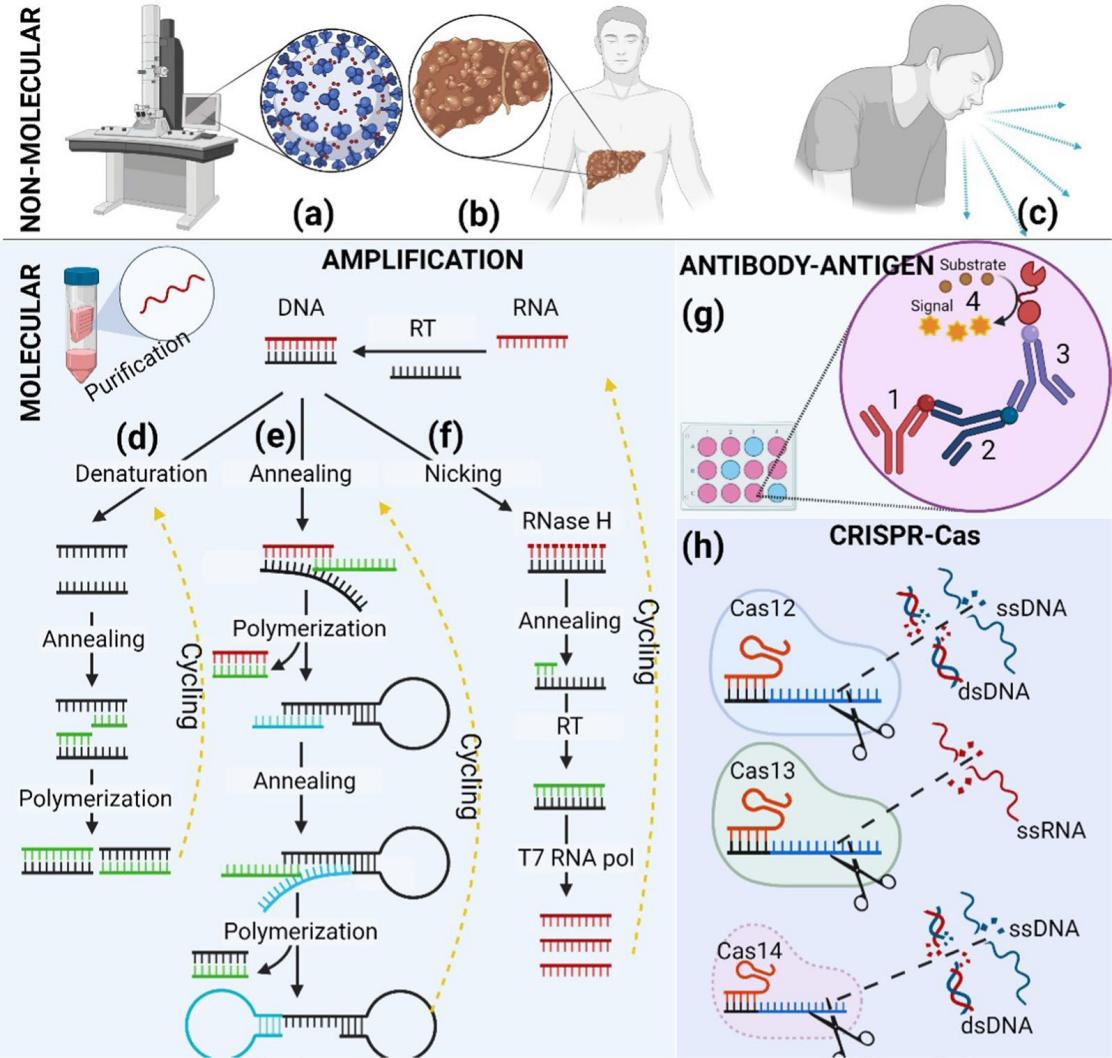
Differences between molecular and non-molecular detection methods. The main application of non-molecular detection is to study the morphology (**a** electron microscopy) of the viruses or to determine (**b** X-ray detection) the symptoms of the viral (**c** visual symptoms detection) disease. However, in molecular diagnostics, a nonspecific reaction (cutting, multiplication, amplification, etc.) or other changes must occur after the target reaction. If the method is not nonspecific, such reactions use additional components that play the role of non-specificity. Also, the purification of nucleic acids is recommended in the use of molecular applications. Nucleic acid amplification increases the amount of DNA or RNA by cyclical repeating of some procedures. In the case of PCR, real-time PCR (**d**), and loop-mediated isothermal amplification (LAMP) (**e**) methods, both a DNA and an RNA fragment can always be used for the analyte, and after amplification, the final product is always a large amount of DNA. However, nucleic acid sequence-based amplification (NASBA) (**f**) applies only to RNA detection, and the uniqueness of the method, that after the cyclic reaction, contains DNA and RNA fragments in the mixture. An enzyme-linked immunosorbent assay (ELISA) is a method that uses a solid-phase-type enzyme-linked enzyme to detect a ligand in a liquid sample using antibodies directed against the protein being measured. The ELISA method is based on a stepwise, sandwich-based combination: 1—capture antibody; 2—antibody detection; 3—secondary antibody conjugation; 4—enzymatic conversion from enzyme to colorimetric or photoluminescence substrate signal. However, fluorescent ELISA usually utilizes secondary antibodies conjugated with a fluorophore. The only drawback is the multilevel application. **h** Cas12, Cas13, and Cas14 are members of proteins used in CRISPR diagnostics. Cas12, Cas13, and Cas14 proteins are on the larger side of the CRISPR diagnostic protein. Cas12 and Cas14 proteins bind directly to the recognized he protospacer adjacent motif (PAM) site and cleave specified DNA sequences. After target cleavage, the Cas12 and Cas14 proteins begin to shred single-stranded DNA nonspecifically. The differences between Cas12 and Cas14—are the size of the protein and RNA length for target binding. The Cas12 protein is 1300 amino acids, and the Cas14 protein is approximately 400–700 amino acids in length. Cas12 DNA targets using 42–44 bp—however, Cas14 protein 140 bp RNA molecules length. The shorter protein spends, the fewer resources are required to obtain the Cas14 protein in the laboratory, and the more extended RNA molecular sequence of the target indicates more excellent fit and accuracy but higher costs. In RNA detection, the technique must combine Cas12 and Cas14-based diagnostics with proteins that convert RNA to DNA. The Cas13 protein directly binds and cleaves specified RNA sequences, and protein can directly detect RNA but not DNA. The Cas13 protein is 1400 amino acids in length, and the RNA guide molecule is relatively short at ~ 64 bp. Thus, more resources are needed to produce Cas13-based detectors, additional Cas13 does not have strict application restrictions, but RNA targets can accept structures that are difficult to cut due to structural limitations. More detailed differences are given in separate article sections and Table 1

Virus detection methods can be divided into two groups (Fig. 3) molecular and non-molecular detection techniques. Assays for developing antibodies against the virus using immunofluorescence or conjugates of immune enzymes, enzyme-linked immunosorbent assay (ELISA), and serological tests, or direct detection of the virus by nucleic acid amplification methods are currently widely used. However, the American Society of Infectious Diseases (IDSA) and World Health Organization (WHO) recommends to apply molecular detection methods for direct virus detection [71], while some other methods also can be used for research, investigation, or the collection of additional domains.

**Table 1:** Differences between molecular detection methods

Method	Molecular methods						
	Nucleic acid amplification			Antibody-antigen detection	CRISPR-Cas		
	PCR, real-time PCR	LAMP	NASBA	Elisa			
Enzyme	DNA polymerase		Reverse transcriptase	Antigen	Cas12	Cas13	Cas14
Analyte preparation	Purification				Purification and amplification		
Target analyte	RNA, dsDNA (direct pathogen)		RNA	Antibody	ss DNA ds DNA	RNA	ss DNA ds DNA
	Direct amount of pathogen			Immune response	Direct amount of pathogen		
Additional components	2 Primers reverse and forward 16–50 bases of complementary nucleic acids; fluorescent reporter and quencher	4 Unique primers (two inner and two outer); fluorescent reporter and quencher	2 set of primers and combination of several modification enzymes; fluorescent reporter and quencher	Conversion of enzyme to colorimetric or photoluminescence substrate signal	For ds DNA targets TTTN site; fluorescent reporter and quencher	Depends on RNA secondary structure; fluorescent reporter and quencher	For ds DNA targets TTTA site; fluorescent reporter and quencher
Non-specific analyte	No	No	DNA	No	ssDNA cleavage	RNA cleavage	ssDNA cleavage
Additional equipment	Thermocycling	No	No	No	No		
Method duration	2–3 h	1 h	2.5 h	7–8 h	2 h		
Method accuracy	99–100	90	93–98	92–98	95–98		

### Non-molecular virus detection methods

The first discovered virus was the tobacco mosaic virus (TMV) in 1882, invented by Iwanowski [72]. He and colleagues showed that extracts of diseased tobacco plants pass through filters that trap bacteria, but the plant still transmitting diseases to other plants. Until 1930 scientists hypothesized that the non-bacterial pathogen of tobacco mosaic disease consists of protein and nucleic acids [73–76]. The first tobacco mosaic virus particles (TMV) were visually identified after the invention of the electron microscope (EM) in 1931 by Ruska [77]. In 1935 the purification and crystallization of the tobacco mosaic virus protein were performed by Stanley [78]. Besides, in 1946 author received a part of the Nobel Prizes in Chemistry award for the crystallization of tobacco mosaic virus [79], that innovation allowed the use of molecular analytical methods. Wendell M. Stanley identified that plant viruses consist of two substances (protein and nucleic acid–RNA) and give high yields of the active virus. High results of active viruses confirm that viral genome replication occurs only within living (host) cells, and studies by other authors have confirmed these claims [80–83]. The visualization of the tobacco mosaic virus was established by X-ray diffraction in 1978 [84–87]. 1960–1990 the nucleic acid (RNA) of the tobacco mosaic virus (TMV) was sequenced [30, 88–93]. Because the tobacco mosaic virus is a single-stranded RNA virus, oligonucleotide primers were used to generate cDNA and encode the RNA sequence. The virus sequencing in 1982 resulted in a fully characterized library of phages, which were later used as a single-chain source for hybridization experiments and site-directed mutagenesis, as well as antiviral techniques. 1989 Powell et al. studies have shown that the accumulation of antisense RNA complementary to the tobacco mosaic virus sequence and the 3’ non-coding region protects the plants from infection [94, 95]. Grafting [96, 97], epigenetics [98, 99], transgenetic [100], and other technologies have been studied to increase the resistance of plants not only to viruses but also to adverse environmental conditions.

#### Electron microscopy based virus detection

Transmission electron microscopy (TEM) is the only technique that can provide simple, fast, and clear morphological images of the virus [101] and differential diagnosis of different agents. The use of electrons for imaging allows a resolution of about 0.2 nm to be achieved, which can be used for visualization macromolecules such as capsid proteins or viral nucleic acids. The electron beams are generated using TEM analysis by either tungsten or LaB6 filament or field emission gun. Monochrome electron beam for biological work in a vacuum accelerates through 40–100 kV voltage and passes through magnetic fields that act as lenses. These electromagnetic fields are generated by solenoids that can concentrate the electron beam. Successful virus diagnosis by transmission electron microscopy is highly dependent on collection methods and sample preparation. Due to the low mass density, the biological structure (carbon, nitrogen, hydrogen) interacts weakly with the electrons used for imaging and therefore has low contrast or detail. TEM’s main advantage is that it creates adequate virus image contrast and resolution. The biological fluids samples can be easily and quickly tested by positive or negative staining. There are two main differences between positive and negative staining. First, the positive staining technique (fixation, post-fixation, embedding resins, multiple staining incubation about four days) is about 500 times longer than the negative staining technique (about 10 min). Second, the final virus image is darker formed against a light background (positive), unlike negative staining when a bright picture of the virus particle is formed against a dark background [102]. The negative staining technique from liquid viral samples, based on the deposition of viruses on carbon-coated grids and stained with heavy metals salts (e.g., lead, tungsten, and uranium ions are used in both staining techniques) used widely. Freezing or formalizing samples is a common mistake in TEM practice [103–106].

The minimum virus concentration required for successful morphologic identification by TEM is about 10^6^–10^7^ particles/ml [107–109]. Since the sample may contain small amounts of virus particles, samples should be concentrated before virus detection to avoid false-negative results [110, 111]. Widely used concentration sampling methods as (i) ultracentrifugation/ultrafiltration [112, 113], (ii) adsorption–elution [114–116]. Differences between concentration methods are detailed by Pasquale et al. [117] The main advantage of the ultracentrifugation method is that it is possible to concentrate all viruses in a sample. The method requires minimal sample manipulation (processed under natural pH), short processing time. Additional benefits are that the ultracentrifugation method achieved higher results than the adsorption-elution method, and equipment is often found in shared laboratories. During one of these procedures, the enrichment viral particles concentration will be approximately 5–100 times higher [118, 119]. Furthermore, virus inactivation, removal of inhibitors, and detection of specific viruses are essential points in detection. The authors did not consider the possibility and combination of using the electron microscopy method and molecular methods.

However, the benefits of TEM in addressing diagnostic problems in clinical virology have been demonstrated in the 1970s and 1980s, when the technique helped discover many clinically essential viruses such as adeno-, entero-, paramyxo-, and retroviruses. Identification and investigation of the morphology (differences in virus size and delicate structure) and the number of total particles (whether or not they are infectious) of all these viruses by TEM helped to more accurate classification of viruses. The main disadvantages of virus detection by the TEM method are particle counting—when counting manually in several grid elements simultaneously, this method is not very accurate. However, by TEM technique can incorrectly identify viruses with similar morphology. However, the advantage of the TEM method is its affordable application to "dirty" clinical samples such as plasma, urine, feces, or to detect the growing virus using cell culture (embryonated egg or laboratory animals). TEM turns out to be necessary to detect and identify a new (a morbillivirus virus [120], swine flu [121], bird flu [122], Ebola virus [123], SARS-CoV-2 [124]) type of viruses. In addition, details of the viral structure may be even more visible if viral preparations are rapidly frozen and vitrified samples are investigated by cryo-EM [125].

TEM analysis has long been a primary quantitative method for counting individual viral virion particles. Advances in microscopy and technology in virology have led to the development of quantitative analysis of viral particles [126]. The technologies include atomic force microscopy (AFM) [127], laser light application multi-angle laser light scattering (MALLS) [128, 129] or nanoparticles tracking analysis (NTA) [130–132] combines resistive impulse sensing (TRPS, method based on Coulter principle) and flow cytometry (FC) variants. The molecular techniques with the advantages of higher sensitivity, no sample concentration, and the ability to quickly process large volumes of samples have replaced TEM in many areas of virologic diagnostics. For virions of viral particles larger than 40 nm, quantitative TEM estimation of viral particles is most commonly used, whereas quantification of viruses of small viruses (less than 40 nm) is often performed using real-time RT-PCR [126, 128, 133]. Real-time RT-PCR results may be incorrect because the number of viral genomes does not necessarily correspond to the number of virions. There are results [109] that show a reasonable accuracy of the TEM quantification method even at a virion size of 30 nm, but this is probably only due to the purification of the virus suspension before the TEM assay.

#### Radiology based virus detection

The use of X-ray microscopy is another powerful alternative to non-molecular virus detection tools based on the high penetration power of soft X-rays in the photon energy range of the water window (photon energy of 250 eV) in hydrated biological material (sample thickness ⩽ 10 microns) [134]. The use of diffraction optics in X-ray microscopes with cryogenic samples allowed the successful imaging of 3-D cells at 20–30 nm resolution [135].

Computed tomography is another tool that describes a fast scan time and a clear image for treating various viral diseases. Unfortunately, there are changes in the organs as the viral illness progresses. In the early stage, pneumonia [136–139] shows several small patches and interstitial changes in the lungs. Liver fibrosis is a disease caused by the hepatitis C virus that complicates all liver processes. Fibrosis results in cirrhosis, which irreversibly destroys the liver [140, 141]. Changes in the liver can also be detected quickly by computed tomography. Examination, detection, and visualization of infected organs by computed tomography have become an indispensable tool for characterizing the early stages of viral infection and monitoring and assessing the severity of the disease. By the way, the clear damaged organ image can also be a significant warning signal for negative results obtained by the molecular virus detection methods (false-negative results).

#### Visual detection of symptoms of viral infection

The simplest way to detect a viral infection is to monitor physiological properties. Fever, dry cough, nasal congestion, runny nose, sore throat, myalgia, and fatigue are the main manifestations of respiratory (SARS-CoV-2 [142, 143], influenza viruses [144–146]). HIV destroys white blood cells (T-lymphocytes) in the human body. T-lymphocytes are responsible for controlling the body's immune system reactions. That means that after an HIV attack in the body, white blood cells can no longer control the body's immune system. The immune system gradually weakens, and the body can no longer defend itself against infections or diseases [147, 148]. As the body weakens, rapid weight loss, muscle aches, joint pain, fever, general weakness, swollen lymph nodes, sore throat, diarrhea, night sweats, rash, drowsiness may occur [149–151]. Chickenpox [152, 153] is an acute viral disease characterized by fever, herpes on the skin and mucous membranes. These first symptoms appear 2 to 3 weeks after infection [154]. However, mention above symptoms can also be caused by different viruses or pathogens, so applying a visual method to make an accurate diagnosis is not appropriate. However, visual diagnosis of the viral disease is widely used to form a preliminary opinion or a starting point for further diagnosis.

### Molecular virus detection methods

Molecular diagnostic methods are used to (i) detect antibodies to different viral proteins or (ii) to directly detect the whole virus or its components. The first molecular diagnostics methods became available in the 1970s when scientists began using recombinant DNA technology (constructed DNA probes) for virus detection [155, 156]. Recombinant DNA technologies: molecular cloning, nucleic acid hybridization, and the use of restriction enzymes to cut DNA at specific sites [157] are the most sensitive in vitro techniques for detecting small viral targets. However, those mentioned above molecular diagnostic methods did not apply to clinical virus diagnostics as the methods were based on radioisotopes (highly ^32^P-labeled nucleic acid probes). These methods were widely used only in virology research laboratories for virus investigation due to their sensitivity.

Detection of viral infection by serological (blood) tests allows the detection of virus type, subtype, viral load (by measuring viral RNA or DNA in the blood), drug resistance, and how long the organism has been infected with the pathogen. Moreover, blood samples can be stored for long periods and remain relatively stable. Plasma or serum is usually stored refrigerated or frozen for long-term storage, and dried blood stains remain stable at room temperature. Many viruses are excreted in large amounts in urine or saliva, so non-invasive samples [158] are also used to detect viruses using molecular virus detection methods. However, the purification of nucleic acid from a sample is the most important step in a molecular method in virology. High-quality nucleic acid is required for most molecular method applications, but they are essential for nucleic acid amplification, antibody-antigen technique, and sequencing. The nucleic acid purification step removes any inhibitors from the sample and concentrating the amount of nucleic acid. Inhibition of PCR and other molecular detection methods by nucleic acid extracts is a well-known phenomenon and has been widely described in several reports. Research has shown that urine [159, 160], phenolic groups (e.g., humic acids) [161, 162], complex polysaccharides (e.g., feces, plant material) [163, 164], collagen (e.g. tissues) [165], heme (e.g., blood) [166–168], humic acid (e.g., soil, plant material) [169–172], melanin and eumelanin (e.g., hair, skin) [173, 174], proteinases (e.g. milk) [175, 176], calcium ions (e.g., milk, bone) [173, 177] adversely negatively affects the test results obtained by molecular detection methods.

#### Nucleic acid amplification methods for the diagnosis of virus induced diseases

Nucleic acid amplification procedures, including PCR, real-time PCR PCR [184], RT-PCR [185], nucleic acid sequence-based amplification (NASBA) [186], and loop-mediated isothermal amplification (LAMP) [187] developed for most respiratory viruses, and today these sensitive nucleic acid amplification methods put to use in a routine clinical or diagnostic laboratory for detection respiratory and other pathogens. Since the development of PCR in 1983 [178, 179] the method was quickly adapted firstly for the detection of the human immunodeficiency virus (HIV) [180–182] and later for the diagnosis of respiratory and other viruses as well [183]. Nucleic acid amplification methods have high sensitivity and strong sequence specificity; the equipment and techniques are also validated [188–190].

Nucleic acid amplification is a straightforward technique for rapidly amplifying billions [191] of DNA copies of interest. PCR or real-time PCR involves effective [192–194] repetitive cyclic polymerization using thermostable DNA polymerase, excess amount of each of the four deoxyribonucleotide triphosphates, two oligonucleotide primers (short strands about 16–50 bases of complementary nucleic acids), and target genetic material. In the first end-PCR point and real-time PCR stage of the cycle, the denaturation of the target double-stranded DNA is performed by heating (at over 90 °C). In the next step, the two primers bind complementarily to two separate single-stranded DNAs (~ 40–50 °C), and at the third stage, the polymerization reaction is performed by DNA polymerase at 60–70 °C. At the end of the first cycle, two double-stranded DNAs are produced from one double-stranded DNA [33, 184, 195–197]. The formula used to calculate the number of DNA copies generated after amplifying a given number of cycles is 2^n^, where n is the number of cycles. Astonishing, using PCR or real-time PCR method, the 105–106 copies of DNR can be made in 25–30 cycles within ~ 2–3 h. In RNA viruses, double-stranded DNA is prepared by cDNA technique using oligonucleotides and reverse transcriptase in the reaction. Real-time PCR is known to be more sensitive, faster, and much easier to perform when compared to conventional PCR by (reduced cycle time and steps, also there is no need for additional electrophoresis, staining, gel documentation, and risk of contamination is reduced. Detailed and comprehensive multiplex (possibilities of identifying multiple infectious agents) real-time RT-PCR study of RNA viruses was performed by Osman et al. The authors [198] used many different fruits and plants virus isolates. The results showed that multiple and single detections of real-time RT-PCR in other virus isolates are similar. The single- and two-stage multiplex real-time RT-PCR assays yielded comparable Cq values. This study has shown that multiple real-time RT-PCR testing simplifies virus detection. The process is partially automated. Due to selected conservative target sequences and RNase inhibitors in the reaction real-time RT-PCR specificity and sensitivity for RNA viruses are 99–100% [199–205].

As mentioned earlier, the nucleic acid amplification technique requires generating and constructing one or two sequences directed to regions of the viral genes or viral structural proteins. Convenient that sequenced viral genomes are publicly available in databases. In molecular genetics, gene sequences contain open reading frames (ORFs) that encode regulatory (e.g., transcription) regions that can potentially be translated into proteins. An ORF is a continuous codon stretch with a start (usually AUG) codon and stops (usually UAA, UAG, UGA) codon [206]. The phylogenetic analysis of open-reading frames (ORFs) sequences identifies and designs highly conservative virus structure regions of interest.

Coronaviruses are large positive-chain enveloped single-stranded RNA viruses, and the 5′-terminal has two open reading frames (ORFs), 1a and 1b, and encodes two large polyproteins that are processed by viral proteases and involved in viral RNA synthesis [207–210]. The study by Udugama et al. showed that coronaviruses have three conserved sequence regions: the RdRP (RNA-dependent RNA polymerase) gene, the E (envelope protein) gene, and the N (nucleocapsid protein) gene [211]. The SARS-Cov-2 virus belongs to beta-Cov lineage B RNA viruses. It is characterized by apparent stick protrusion [212, 213] on the virus's surface and an abnormally large RNA genome [214]. SARS-Cov-2 virus genome encodes spike (S) protein, matrix protein (M) nucleocapsid (N) protein, and envelope (E) proteins. However, the RdRP (3.6 copies/reaction) and E (3.9 copies/reaction) genes sequence have threefold higher analytical sensitivity than the N (> 10 copies/reaction) gene [215–217] and are, therefore, highly recommended to use as targets for direct detection of coronaviruses. However, knowing the capabilities of PCR and real-time PCR technology, multiplex PCR can be used for three or more gene sequences in a single sample. Recently, multiplex RT-PCR studies have described the evaluation of different viruses and mutations [218–221].

HIV and other retroviruses are also RNA viruses and have three main open reading frames (ORFs). First, a gag or group-specific antigen is the major structural protein. The second ORF is pol, encoding a polyprotein containing reverse transcriptase, integrase, and protease precursors. The third–env–encodes glycoproteins that are displayed on the virus's surface and cause viral recognition and fusion with the host cell [222]. Corti et al. confirm that the HIV gag and pol gene regions are excellent targets for direct virus detection. However, the env gene region is suitable as a target for antibody detection [223].

Influenza A, B, and C are negative-sense, single-stranded, segmented, and enveloped RNA (approximately 100 nm in diameter, 12,000–15,000 nucleotides) viruses that belong to the orthomyxoviridae family. The influenza virus genome contains 7–8 single-stranded RNA gene segments (8 segments have influenza virus A and B, 7 parts—virus C) that encode ten different proteins. Matrix protein (MP), hemagglutinin (HA), neuraminidase (NA) proteins are present on the influenza virus surface and play essential roles in infecting a host cell. Hemagglutinin protein (HA) and neuraminidase protein (NA) are grouped into subtypes 16 and 9, and the sequences of these subtypes are very different even in subspecies, making them an effective means of monitoring viral changes. The influenza virus is unique due to mutations that lead to changes in two external proteins, HA and NA. Currently, antibodies to the NP gene are the sequence of the target molecule to detect influenza. Hemagglutinin (HA) gene and neuraminidase (NA) gene sequences are the most critical antigenic sites for producing a protective immune response. Matrix protein (MP) genes for influenza A and B viruses, hemagglutinin (HA) gene sequences for influenza C virus were used as targets for PCR primers as well as other nucleic acid amplification methods in 1991 [224]. A comparative study has also been performed on the PCR system by varying the length of the forward and reverse primers (21 and 32 nucleotides in length) and the calcium chloride (10–200 mM) concentration [225]. The authors declare that the reaction's 90 mM of calcium chloride is 10–100 times more sensitive than the proposed antigen immunoassay system.

By Starick et al., the influenza A virus RNA was purified/prepared by the acid guanidine-thiocyanate method [226], hemagglutinin (HA) part selected nucleotide sequence for RT-PCR [227]. The authors didn’t detail the concentration of viral RNA used. DNA fragments were evaluated after the PCR reaction visually in agarose gel. PCR products from selected isolates with known sequences were sequenced after cloning to confirm that accurate amplification was achieved. Paramyxoviruses of serotypes were used as controls to ensure the specificity of the studies. Hence, PCR methods have demonstrated no false-positive results. For further investigations by other authors [29, 228, 229] the same RNA purified samples were chosen and more conserved gene sequence (matrix protein (MP)) as target primer for PCR reaction for direct virus detection. After electrophoresis, agarose gel visually assessed the PCR product (212 bp for influenza A virus, 365 bp for influenza B virus). The results of these studies clearly showed high sensitivity and suitability for virus detection in a PCR reaction.

Spackman et al. were developed a real-time RT-PCR assay based on matrix protein (MP) gene sequence as target primer [230]. The authors declare that the detection limit of the matrix gene real-time RT-PCR assay is 10 fg or approximately 1000 copies of the target RNA. The matrix protein (MP), hemagglutinin (H), and neuraminidase (N) gene sequences, and the TaqMan probe with different FAM, VIC, and NED fluorescent signals have been experienced for single-step real-time RT-PCR assay by Payungporn et al. [231]. Payungporn et al., Panning et al. studies of a wide range of influenza A viruses have shown that the MA and HA gene sequences are equally susceptible to virus detection and are both suitable for real-time PCR [232]. The limit of detection with FAM fluorescent signals reached 384 RNA copies/ml. Multiplex [233] real-time RT-PCR analyses are increasingly used in clinical in vitro diagnostics to detect seasonal influenza viruses. Although PCR shows excellent sensitivity [234], it has several drawbacks: expensive reagents and devices, complicated operation, and reverse transcription for RNA detection.

Loop-mediated isothermal amplification method (LAMP) [235]—for DNR amplification at single incubation temperature using a set of four (two inners and two outer) unique primers to recognizing eight distinct sites of the target sequence. A DNA-displacing DNA polymerase (Bst or phi29 DNA polymerase) is used to initiate the synthesis, and the two primers form loop structures to facilitate and accelerate further amplification steps. RT-LAMP has been used for rapid and straightforward detection of RNA (10 copies/reaction) viruses [236]. The test results are analyzed with the naked eye due to the fluorescent dyes present in the system. Tracking results in real-time is also possible. Numerous [215, 237–240] studies have demonstrated the successful use of various LAMP tests to detect coronavirus RNA in serological samples. However, the LAMP method has a drawback—the complexity of the methodology. The method requires the ability to design complex primers, and the method's resolution, accuracy, or specificity depend on this step.

NASBA—self-sustained and repetitive sequence replication and transcription reactions are very similar to PCR or real-time PCR techniques because of oligonucleotide primers. A multilevel and complex isothermal method is suitable for detecting RNA viruses: HIV [241–243], coronaviruses [244, 245]. Several modification enzymes (AMV reverse transcriptase, ribonuclease H, and T7 RNA polymerases) and their activities combine to produce many copies of double-stranded DNA and new RNA targets for further application. The method can make 107 copies of nucleic acid targets within 2.5 h [246, 247]. However, the technique is challenging. The oligonucleotide sequence must be complementary to the target virus RNA and the T7 RNA polymerase promoter sequence; otherwise, the amplification reaction will stop. The T7 RNA polymerase can amplify up to 50–1000 new RNA copies by transcription from one copy of RNA. Since this is a spontaneous and continuous process, the reaction ends when all the components are agitated, and equilibrium is reached without any thermocycling at constant 41 °C temperature. Of course, the fluorescent label can monitor amplification in real-time and obtain rapid test results [241]. The advantages of this method are two. The final reaction products are both dsDNA and RNA. DNA is the most often chosen for further application because it is more stable to various environmental conditions. However, the method's reliability for the detection of HIV in the *gag* and *pol* regions of the genome [248–250] is 93–98%.

All the nucleic acid amplification methods listed here are widely used for detecting different viruses in samples. However, they are also commonly used in the application by combining other regions of the genome sequences to see individual virus prototypes [251] to increase the sensitivity and accuracy of the method.

A slight improvement in RT-PCR allowed the discovery of the Abbott ID Now COVID-19 method. The Abbott ID Now molecular point-of-care COVID-19 detection test is associated with high sensitivity and specificity, providing reliable positive results in 5–13 min [252, 253]. Abbott ID Now, the technique is based on isothermal nucleic acid amplification by using primers to allow specific amplification of RdRp viral target with a claimed LOD of 125 copies/mL [254]. Evaluation of the Abbott ID Now method has shown that the study performs well on strong and moderately positive samples but dramatically reduces the sensitivity to weakly positive models, confirming the findings of other published studies [255]. Therefore, Abbott ID Now has not been FDA cleared or approved.

#### Antibody-antigen complex detection-based methods for the determination of virus induced diseases

Conventional virologic methods are well known established [256]. The culture of the virus with immunohistological confirmation of viral antigen has long been the standard virus detection method. Many cell culture species exist for influenza viruses, such as human adenoid primary epithelial cells, Vero cells, MRC-5, Madin–Darby canine kidney (MDCK) cell line, and primary monkey kidney cells [257]. In clinical or diagnostic laboratories, after 2–10 days, the viral culture is performed by immunological methods, such as a direct immunofluorescence method or molecular biologic methods [258].

The hemagglutination inhibition (HAI) [259] method titrate the antibody response to a viral infection. The HAI assay is based on the virus's ability to hemagglutinate (bind) red blood cells, forming a 'lattice' and preventing red blood cells from clumping. However, the greatest stringency of the method is sensitive to some inhibitors, and in addition, not all viruses have the ability to hemagglutinate in interaction with antibodies. Rubella [260], avian influenza [261], influenza A and B [262, 263], swine [264], and other viruses have the properties listed above.

Directing is an enzyme-linked immunoassay (EIA) membrane method performed in a solid membrane based on an enzymatic reaction between enzyme-conjugated monoclonal antibodies specific towards virus proteins. Then the captured virus antigen–antibody pair is visualized by an enzymatic color development reaction [265, 266]. The publications on EIA state that the method has high sensitivity, high specificity (nucleoproteins are used as antigens), rapid diagnosis (analysis last less than 15 min), and technical simplicity. This method is applied prospectively directly, qualitatively, simultaneously for the detection of A and B virus antigens at the same time in different clinical samples of symptomatic patients [267]. However, comparing the data between the results of the cell culture method (shell vial) and the EIA method showed that the overall sensitivity of the EIA method is only 68.9%. Hence, the EIA is one of the traditional methods for detecting type A and B influenza viruses in clinical diagnosis.

In the case of COVID-19 after 14 days [268], direct SARS-Cov 2 virus detection from the respiratory tract is not available (false-negative results), but antibody detection is possible and recommended. Recent preliminary studies have shown that both IgM and IgG increase with the SARS-Cov-2 infection phases and reach the highest level between two and three weeks [269, 270], even after a month of IgM traces are found [271]. Antibody studies focus on immunogenic coronavirus proteins: S protein, most exposed to the viral protein, and N protein, which is abundantly expressed during infection [272]. Since respiratory disease is also a consequence of coronavirus, researchers determined IGA levels in patients with and without SARS-CoV-2. Although a strong IgA response was observed in patients with COVID-19, immunoglobulin peaked within two weeks. The IgA-based immunoassay [273] is less specific than the IgG-based ELISA due to the likelihood of samples infected with other coronaviruses.

In the case of HIV, another assay for virions, the enzyme-linked immunosorbent assay (ELISA) detecting HIV-1 gag, p24, has low sensitivity (103 to 106 virus particles/ml) and can also detect virus-like particles lacking genomic RNA and p24 released from dead cells [274–276].

For the semi-quantification of immunoglobulin G (IgG) antibodies in human serum, the lateral flow point-of-care techniques with specially designed recombinant viral proteins are increasingly started to use. The point-of-care system combines a biotinylated aptamer with a streptavidin test line and a secondary antibody control line. Rapid antibody tests are available, but the method cannot use them to monitor viral progression. Recent works with the Ebola virus [277], influenza virus [278, 279], HIV [280–282] have shown that the lateral flow point-of-care technique can achieve a significantly improved limit of detection and multiplex detection. The capsid protein (p24 antigen) and anti-HIV antibodies are the primary viral markers used to detect HIV infection in the lateral flow point-of-care system. The lateral flow point-of-care system was designed to detect amplified (142 bp) HIV RNA quantitatively. When RNA is distributed on a conjugate strip, the RNA binds to complementary oligonucleotides conjugated to gold nanoparticle probes. However, most studies suggest that in combination with nucleic acid amplification methods (NASBA, RPA, etc.), a lateral flow monitoring test can detect RNA concentrations of HIV or other viruses [280, 281, 283] But integrating lateral flow point-of-care analysis with amplification and sample preparation technologies complicates the application of the method in clinical diagnostics due to complexity.

Enzyme-linked immunosorbent assay (ELISA) [284] technique is used to quantify proteins, peptides, antibodies, or hormones in a biological system. The amount of antibodies in the blood reflects the body's overall ability to protect itself against infections and its ability to form an immune system. Antibody changes can occur in a variety of autoimmune diseases. In total, the human body produces five known classes of antibodies [285], labeled Ig (meaning immunoglobulins), which belong to A, M, G, E, and D classes. Thus, participants in the primary, acute infectious process are class M immunoglobulins (IgM), showing a critical phase. Subsequently, IgM antibodies in the blood disappear, and class G immunoglobulins (IgG) production begins. In terms of IgE, it is used to diagnose various parasitic invasions and allergic reactions, and IgD performs an ancillary function. ELISA is a sandwich method [286–288] in which the antigen of a specific pathogen is layered on the surface, a biological substance is added (the patient's blood serum), and immune complex formation is formed. The assay is fixed with a unique chromogen component, and the color change is visually observed with fluorimeters. The relative disadvantage of ELISA is that the method only detects the immune response—antibodies but does not detect the pathogen itself.

Conservative sequences of the viral envelope genome are constructed for the ELISA method, which aims to detect antibodies to the virus formed in the body. Antibodies to the HIV envelope proteins *gp120* and *gp160* [289] are the most commonly detected in all saliva samples taken from HIV-positive individuals. Antibodies to other viral proteins (*p65, p51, gp41, p35, p24, p18*) are detected in saliva at random without a clear correlation [290] with the clinical stage of the disease. Due to the growing global demand for rapid tests, attempts have been made to develop and evaluate an IgG-based ELISA for COVID-19 for detection. To determine the concentration of antibodies (anti-SARS-CoV-2), which belongs to the IgM and IgG class of proteins and these anti-SARS-CoV-2 antibodies selectively recognizing coronavirus spike (S) and nucleocapsid (N) proteins [291–293]. Studies show that in COVID-19, the positive IgG level is reaching 100% approximately 20 days after the onset of symptoms [294]. The mean seroconversion day for both IgG and IgM was 13 days after the onset of symptoms. IgM seroconversion occurred at the same time, earlier or later than IgG [26, 295, 296]. ELISA for COVID-19 detection was found to be 92.37% sensitive and 97.9% specific. The results show that the actual status of the infection and its spread can be determined using IgM and IgG antibodies against SARS-CoV-2 in serological tests such as ELISA. Furthermore, the ELISA method was developed and validated to detect anti-SARS-CoV-2 human IgG antibodies. Proposed ultraviolet colorimetric assay method—magnetic nano-enzyme linked immunosorbent assay (MagLISA). Here the [297–303] authors combined a silica shell with magnetic nanoparticles (MagNBs) and gold nanoparticles for influenza A virus (0.02 pg/ml) monitoring. The main concepts of the developed sensing platform for rapid and sensitive detection of influenza A virus are two different probes capable of specifically recognizing the target virus. Anti-influenza virus antibody was immobilized on positively charged AuNPs via electrostatic attraction (fixation probe). Monodisperse Fe_3_O_4_ and nanocluster (FNC) modified with a silica coating were chosen as the capture probe to inhibit the activity of enzymes from the iron oxide surface (Mag). The fixation probe recognizes the target virus by a specific antigen–antibody interaction, after which the antibody-antigen structures are assembled while applying a magnetic field. Specific antigen–antibody or receptor-ligand interaction can be determined by various physicochemical methods ranging from micromechanical [304], gravimetric [305, 306], optical [307, 308], surface resonance [309–311], ellipsometric and electrochemical [312–314].

Electrochemical sensors sometimes are less sensitive in comparison to some other analytical signal detection methods mentioned above. However, they enable the determination of antibodies against some virus-proteins or vice versa—virus-proteins in somewhat turbid samples [312–314] so the method has not been commercialized yet. Diseases caused by viruses and other pathogens can be diagnosed using immunosensors. Fluorescence is a sensitive analytical signal determination method that is promising when low analyte concentrations are present in the aliquot [307, 308, 315]. However, in most photoluminescence-based ways, additional photoluminescence probes should be applied, which sometimes is a significant drawback because they need additional expensive chemicals such as antibodies conjugated with photoluminescence probe [314–316]. This drawback can be avoided when Surface Plasmon Resonance (SPR) based sensors [309–311, 317] and spectroscopic ellipsometry [318–325] based methods are applied. In addition, these sensors are less sophisticated in comparison with more sensitive optical methods. The most innovative electrochemical techniques, such as scanning electrochemical microscopy, also can be used for immunoanalytical purposes [312]. It should be noted that some resonance techniques (e.g., quartz crystal microbalances QCM and especially QCM with dissipation QCM-D) [305] and electromechanical resonators such as capacitive micromachined ultrasound transducers (CMUT) [304, 326] based method can be also well exploited in the development of immunosensors. Very promising are forms based on the application of molecularly imprinted polymers (MIPs), while virus proteins can be imprinted within some electrochemically deposited conducting polymers [34, 327]. Recent developments in protein imprinted MIP sensors are overviewed in other review papers [328]. For better validation or measurement results combination of very different analytical methods can be performed simultaneously [317].

#### CRISPR-Cas system-based determination of viral infections

Specific High-sensitivity Enzymatic Reporter un-LOCKing (SHERLOCK) and DNA Endonuclease Targeted CRISPR Trans Reporter (DETECTR) are diagnostic tools that are based on the CRISPR-Cas system and can be used to detect specific RNA or DNA at low attomolar concentrations [329]. CRISPR (Clustered Regularly Interspaced Short Palindromic Repeats) is a natural tool for genome editing that is part of the prokaryotic immune system used to fight viruses and immunize the organism [330–335]. Cas (CRISPR-associated protein) in the CRISPR system is guided by RNA to target place, and Cas proteins are endonucleases or ribonucleases, an enzyme that cuts DNA or RNA [336–341]. Thus, the CRISPR-Cas complex acts as an antivirus system Cas protein programmed by guide RNA to cleave specific target viral DNA or RNA [336–340]. Over the past few years, researchers have realized that they can use the CRISPR-Cas system to cleave any DNA or RNA at the site of interest by replacing the guide RNA or Cas protein in the system [342, 343]. The nucleases encoded by CRISPR-Cas can accurately recognize nucleic acid sequences. Therefore, their use in diagnostics for rapid testing is being investigated worldwide. The methods developed are sensittive, capable of rapid detection, do not require expensive equipment or training, and can be performed point-of-care.

Gootenberg et al. 2018 developed a method for a multiplexed and portable nucleic acid detection platform [343–345]. SHERLOCK technique is a detection method that combines isothermal amplification with a CRISPR Cas13 cleavage system. Although either Cas12 or Cas13 enzyme [346–349] can be used for nucleic acid detection, SHERLOCK protocol focuses on Cas13 because it shows the highest sensitivity in the application. The technique requires introducing the T7 RNA polymerase promoter during pre-amplification and T7 RNA polymerase during the detection reaction to generate RNA and for Cas13 trans- cleavage activation. Cas13a protein guided by RNA binds and cleaves target RNA. SHERLOCK has become an excellent tool for detecting RNA viruses due to non-specific cleavage [350, 351]. This method has advantages such as no additional cDNA step is required, the short nucleotide sequence fused to a fluorescent reporter, and the quencher as point readings or in real-time with a plate reader or other compatible fluorimeter. The system is rather complex, and the construction of primers must be complementary for the virus and the enzyme sequences used in the system. However, the method requires several additional amplification steps, making it difficult to use the CRISPR-Cas technique in clinical diagnostics.

To detect the SARS-CoV-2 virus, the guide RNA is constructing according to the conservative sequence of the neuraminidase (N) gene, spike (S) gene, and replicase polyprotein 1ab (Orf1ab) gene [352, 353] under both lateral-flow strip and fluorescence readouts according to varying nucleotide lengths (7–40 bp). It has been observed that low sensitivity using N genes is probably due to the longer (> 28 bp) generated N RPA amplicon [354, 355]. If, by extending the response time of the N gene RPA to 1 h, the sensitivity of detection increases and corresponds to the sensitivity of the S gene. Using lateral-flow protocols 10^4^–10^5^ copies/ml [28, 356] sensitivity is achieved. Alternative amplification methods have shown that the N gene has a higher copy number than other segments of the SARS-CoV-2 genome, which helps to increase the sensitivity of detection. With the SHERLOCK platform, 1 copy/ul of Zika virus (ZIKV) and dengue virus (DENV) can be detected directly from patient samples (e.g., serum, urine, and saliva) within 2 h [357] and have successfully used the system for the detection of human viruses.

Chen et al. reported [358] the DETECTR (DNA endonuclease directed CRISPR trans reporter) method based on the CRISPR system and provides a straightforward platform for molecular diagnostics. DETECTR, like SHERLOCK, also achieves atomic sensitivity [347, 359]. DNA is detected by combining activation of Cas12a or Cas14a single strand DNase nonspecific single strand deoxyribonuclease with isothermal amplification, which allows rapid and specific virus detection from patient samples. Cas12a-DETECTR [360, 361] platform guided by RNA detect and cut target double-strand DNA by cis cleavage, also have the additional ability for trans- single-stranded DNA cleavage. Cas14a-DETECTR [362] mechanism guided by RNA binds and cut single-strand DNA. However, additional steps are required: cDNA (for RNA viruses) in the case of Cas12a protein, double-strand DNA conversion to single-strand DNA in case of Cas14a, and the nucleotides are designed according to the target RNA sequence and a short nucleotide sequence fused to a fluorescent reporter and quencher. Using lateral-flow protocols 10^4^ copies/ml sensitivity is achieved. Primers targeting the SARS-CoV-2 genes E (envelope) and N (nucleoproteins) were designed [363] for the Cas12a-DETECTR. SARS-CoV-2 virus RNA is purified from the patient sample, followed by reverse transcription and isothermal amplification using loop-induced amplification (RT-LAMP) to generate cDNA [27]. Detection of nucleic acid is performed under both lateral-flow strip and fluorescence reading conditions. Comparison of SARS-CoV-2 DETECTR lateral-flow strip with real-time RT-PCR methods showed 95% and 100% accuracy, respectively [27].

## Conclusions

Nucleic acid amplification assays are described as a ‘Gold Standard’ method because of the general-purpose reagents manufactured and approved according to International Organization for Standardization (ISO) normative compliance and quality assurance (QA) rules for the accuracy of the method. Therefore, the regulatory framework and the validity of the technique ensures the reproducibility of the results, and the method is still widely used in the study, research, investigation, and detection of viruses and other pathogens. However, the new generation analytical method based on the nonspecific DNA cleavage observed in CRISPR-Cas type II (Cas9), V (Cas12 and Cas14), and VI (Cas13) systems provide promising advances in CRISPR-based diagnostics of emerging infectious diseases. Already developed and commercialized SHERLOCK and DETECTR methods are straightforward to apply for the detection of new pathogens. However, separate parts of the diagnostic procedure (nucleic acid amplification and detection, signal detection) as in nucleic acid amplification assays are necessary, and a more significant signal is obtained by more straightforward extraction of RNA from body fluid directly. These two advanced methods are based on thorough validations and field tests of diagnostic techniques to guarantee their functionality and ensure that virus or other pathogens detection is susceptible, high quality, specific, and well-functioning. Besides, the sensitivity and accuracy of the methods depend only on the chosen virus ORF gene sequence region. Hence it is worth picking several specific sequences in ORF-region for multiplex assay to quickly and accurately diagnose viral infection. However, the diagnosis of viral infections still faces many obstacles. Therefore, the knowledge and clarity about the methods will facilitate progress in this area.

## Data Availability

Not applicable.
